# Simultaneous transcatheter edge-to-edge repair (TEER) for severe mitral and tricuspid regurgitation is feasible, safe, and associated with good clinical outcome

**DOI:** 10.1371/journal.pone.0339837

**Published:** 2026-02-10

**Authors:** Johannes Gollmer, David Zweiker, Alexander Peikert, Viktoria Santner, Ivan Vosko, Nicolas Verheyen, Klemens Ablasser, Ewald Kolesnik, Nora Schwegel, Birgit Zirngast, Wolfgang Marte, Günther Laufer, Daniel Zimpfer, Martin Andreas, Eva Buschmann, Gabor G. Toth, Heiko Bugger, Andreas Zirlik, Albrecht Schmidt

**Affiliations:** 1 Department of Cardiology, University Heart Center Graz, Medical University of Graz, Graz, Austria; 2 3rd Medical Department for Cardiology and Intensive Care, Klinik Ottakring, Vienna, Austria; 3 Department of Cardiac Surgery, University Heart Center Graz, Medical University of Graz, Graz, Austria; 4 Department of Anesthesiology, Medical University of Graz, Graz, Austria; 5 Department of Cardiothoracic Surgery, Medical University of Vienna, Vienna, Austria; Showa University: Showa Daigaku, JAPAN

## Abstract

**Background:**

Mitral regurgitation (MR) and tricuspid regurgitation (TR) commonly coexist in patients with heart failure (HF). Their concomitant occurrence carries a much poorer prognosis than isolated valve disease. Transcatheter edge-to-edge repair (TEER) of MR and TR is safe and effective, but there is limited data on combined MR/TR TEER.

**Objective:**

The study evaluates the safety and efficacy of combined TEER for MR and TR in a real-world cohort.

**Methods:**

This retrospective safety and efficacy analysis included the first 40 patients treated with combined MR/TR TEER between 2019 and 2021 at our single tertiary care referral centre.

**Results:**

Combined procedural success (MR reduction ≥2° and TR reduction ≥1°) was achieved in 80% of the cases. Simultaneous TEER was safe, with no intraprocedural death, myocardial infarction (MI), stroke, or major bleeding. At 1-year follow-up, the median New York Heart Association functional (NYHA) class improved by one grade; twelve patients (30%) died, and fourteen patients (35%) were hospitalized for HF. Procedural success and postprocedural residual MR ≤ 1° were associated with reduced 1-year mortality rates but not HF hospitalizations.

**Conclusion:**

Combined MR/TR TEER is safe and reduces MR and TR in most patients, conferring a potential benefit regarding symptoms and prognosis. Randomized controlled trials (RCTs) are needed to rigorously evaluate combination therapy in this setting.

## 1. Introduction

Mitral regurgitation (MR) and tricuspid regurgitation (TR) are both common pathologies frequently coexisting in patients with heart failure (HF). Recent data from the ESC-HFA Heart Failure Long-Term registry demonstrate a prevalence of 11% of combined moderate to severe MR and TR in patients with HF [1]. Upon differentiation for ejection fraction (EF), this disease pair occurred in HF patients with preserved EF (HFpEF, defined as an EF above 50%) in 8%, in those with mildly reduced EF ranging from 40% to 50% (HFmrEF) in 7%, and in those with reduced EF (HFrEF, defined as an EF below 40%) in 12%. Patients with both MR and TR afford an inferior prognosis, with risks clearly surpassing those of patients with MR alone [1,2]. Transcatheter edge-to-edge repair (TEER) has become widely utilized to treat patients with chronic HF and MR or TR. Evidence from randomized controlled trials (RCTs) evaluating this treatment suggests benefits in survival and reduced HF hospitalizations for selected patients with secondary MR [3]. For severe TR, RCTs indicate at least a symptomatic benefit for patients treated with TEER [4].

Real-world evidence from several registries corroborates a robust symptomatic benefit for patients treated for either MR) or TR) with a TEER) procedure. If both valves are affected and a surgical approach is selected for MR, the European Society of Cardiology (ESC) guidelines recommend concomitantly treating severe secondary TR. A simultaneous surgical approach should be considered even in cases of mild-to-moderate TR with a dilated annulus [5]. In line with these surgical recommendations, recent interventional data suggest that only one-third of patients with significant MR and measurable TR show a reduction of TR by ≥1° following TEER of severe functional MR, while 19% even experienced worsening of the underlying TR [6]. Therefore, concomitant interventional repair of both valves might be a therapeutic option for patients suffering from both MR and TR. So far, few studies have reported data on the safety, effectiveness, and clinical outcomes of concomitant TEER addressing MR and TR [7,8].

## 2. Methods

### 2.1. Patient selection

This retrospective analysis included the first 40 consecutive patients receiving a concomitant TEER for MR and TR at the University Heart Center Graz from April 2019 to November 2021. During this period, all patients with concomitant severe mitral- and tricuspid regurgitation were considered for concomitant repair if there was no evidence of right heart failure or severe pulmonary hypertension. All patients treated with concomitant repair had been deemed eligible for the procedure by the local interdisciplinary heart team. All procedures were performed using transcatheter edge-to-edge repair systems provided by Abbott. For mitral valve interventions, various generations of the MitraClip system (including XTR, NT, and later G4 devices) were used according to anatomical suitability. Tricuspid valve repair was initially performed using adapted MitraClip systems. From July 2020, the dedicated TriClip system was employed for all tricuspid interventions. In all cases, the mitral valve was treated first, followed by the tricuspid valve within the same procedure. The study design was reviewed and approved by the local ethics committee (33–505 ex 20/21). Due to the retrospective nature of this analysis, local ethics committee waived the need for informed consent.

### 2.2. Data collection and endpoints

Data collection for this manuscript was conducted between 10/07/2021 and 01/02/2023. Baseline characteristics were assessed by reviewing the hospital’s clinical information system (HIS), which incorporates the clinical documentation of all state hospitals in Styria, covering 85% of hospital admissions in this region. Pre- and postprocedural echocardiographic parameters were obtained by reviewing digitally stored echocardiographic loops by two experienced blinded echocardiographers. According to current recommendations, the baseline and postprocedural severity of MR and TR were determined with an integrative approach [9]. An extended grading system with five severity grades was used for TR [10]. Procedural characteristics were extracted from the procedural reports, and complications were assessed by reviewing the documentation in the clinical information system. Procedural success was defined as MR reduction ≥2° and TR reduction ≥1°. NYHA status before intervention and at one-year follow-up was extracted from the HIS. Hospitalizations for HF (HFH) were also derived from the HIS. After one year, mortality was assessed by interrogating the national statistical Austrian database Statistic Austria, where all deaths in Austria are documented by date. HFH and 1-year Mortality were compared between patients with procedural success, patients with residual MR > 1° vs. 1° or less, and patients with residual TR > 1° vs. 1° or less, and combinations thereof. During data acquisition in the way mentioned above, the authors could identify individual participants. After exportation data was anonymized and individual participants could no longer be identified.

### 2.3. Statistics

Categorical variables were expressed as counts (percentages), and continuous variables were shown as mean ± standard deviation (SD) in the case of normal distribution. Non-normally distributed continuous variables were reported as median with interquartile range (IQR). Kaplan-Meier analysis was used to estimate the cumulative incidence of clinical endpoints, and differences between groups were calculated using the log-rank test. Associations of procedural outcomes with clinical events were assessed by Cox proportional hazards models with and without adjustment for a propensity score predictive for overall procedural success, residual MR ≥ 1°, or residual TR ≥ 1°, respectively. Propensity scores were based on multivariate logistic regression models, including baseline covariates for age, sex, body mass index (BMI), type 2 diabetes mellitus, hypertension, atrial fibrillation, ischemic heart disease, and EF, selected as typical confounders for adverse outcomes. P-values of <0.05 were considered statistically significant. All analyses were exploratory. No formal correction for multiplicity was applied, and p-values are reported descriptively. Emphasis was placed on effect sizes and corresponding 95% confidence intervals rather than on dichotomous significance testing. Statistical analyses were performed using IBM SPSS Version 27 (IBM Corp) and Stata 17.0 (StataCorp).

## 3. Results

### 3.1. Baseline characteristics and echocardiographic features at baseline

The median age was 78 years (± IQR 10), with a mean BMI of 25 kg/m^2^ (± SD 4); 60% of the treated patients were female. Most patients were highly symptomatic, with 80% classified as NYHA III or IV. Ischemic heart disease was present in 40% of the patients, while dilated cardiomyopathy affected 32%. A history of atrial fibrillation or flutter was noted in 93% of the patients, and intracardiac leads were present in 30%. 85% of the MRs and all TRs were classified as functional. The mean EF was 45%, with 57% of patients having an EF < 50% and 43% having an EF > 50%. Right heart parameters showed right ventricular and right atrial dilatation, along with mildly reduced right heart function. Baseline characteristics are reported in Table 1.

**Table 1:** Baseline characteristics.

Variable	Overall population (n = 40)	No procedural success (n = 8)	Procedural success (n = 32)	P-value
Age at procedure – years	78 (73 - 83)	79.5 (77–85)	77.5 (73–82)	0.28
Female sex – n (%)	24 (60)	5 (62.5)	18 (56)	0.75
Height – cm	169 ± 8	166 ± 5	169 ± 8	0.25
Weight – kg	70 ± 13	66 ± 13	71 ± 13	0.35
BMI – kg/m^2^	25 ± 4	24 ± 4	25 ± 4	0.67
NYHA – n (%)				0.95
II	8 (20)	1 (12.5)	7 (22)	
III	29 (72.5)	7 (87.5)	22 (69)	
IV	3 (7.5)	0	3 (9)	
**Comorbidities – n (%)**				
Ischemic Heart Disease – n (%)	16 (40)	4 (50)	12 (37.5)	0.52
Dilated Cardiomyopathy – n (%)	13 (32.5)	0	13 (41)	0.028
Diabetes mellitus – n (%)	8 (20)	2 (25)	6 (19)	0.69
Arterial Hypertension – n (%)	33 (82.5)	8 (100)	25 (78)	0.15
Hypercholesterinemia – n (%)	15 (37.5)	2 (25)	13 (41)	0.41
COPD – n (%)	8 (20)	1 (12.5)	7 (22)	0.55
CKD (≥ IIIa) – n (%)	33 (82.5)	7 (87.5)	26 (81)	0.85
Atrial fibrillation/flutter – n (%)	37 (92.5)	7 (87.5)	30 (94)	0.55
Pulmonary Hypertension – n (%)	15 (37.5)	2 (25)	13 (41)	0.41
PAD – n (%)	6 (15)	2 (25)	4 (12.5)	0.38
Cerebrovascular artery disease – n (%)	1 (2.5)	1 (12.5)	0	0.04
Previous myocardial infarction – n (%)	5 (12.5)	0	5 (16)	0.23
Previous PCI – n (%)	12 (30)	2 (25)	10 (31)	0.73
Previous CABG – n (%)	4 (10)	0	4 (12.5)	0.29
Previous valve surgery – n (%)	3 (7.5)	0	3 (9)	0.23
Implanted intracardiac Device – n (%)	12 (30)	1 (12)	14 (43)	0.61
Pacemaker – n (%)	7 (17.5)	1 (12.5)	6 (19)	0.68
ICD – n (%)	4 (10)	0	4 (12.5)	0.29
CRT – n (%)	1 (2.5)	0	1 (3)	0.61
Previous Stroke/TIA – n (%)	3 (7.5)	0	3 (9)	0.37
**Baseline Laboratory values**				
Creatinine – mg/dl	1.35 (1.09–1.73)	1.29 (1.04–2.36)	1.39 (1.09–1.71)	0.855
Hemoglobin – g/dl	12.0 ± 1.7	11.7 ± 1.8	12.2 ± 1.7	0.48
NT-proBNP - pg/ml	3891 (2356–9442)	4281 (2539–12525)	3666 (2356–9442)	0.93
hsTroponinT – pg/ml	37 (20–68)	42 (24–123)	37 (19–64)	0.58

Continuous variables given as median [25th-75th percentile] or mean ± standard deviation and counts as absolute frequencies (column%). Procedural success was defined as reduction of MR ≥ 2° and reduction of TR ≥ 1°.

Abbreviations: BMI = body mass index; CABG = coronary artery bypass graft; CKD = chronic kidney disease; cm = centimetre; COPD = chronic obstructive pulmonary disease; CRT = cardiac resynchronization therapy; ICD = implantable cardioverter defibrillator; kg = kilogram; NT-proBNP = N-terminal pro-B-type natriuretic peptide; NYHA = New York Heart Association; PAD = peripheral arterial disease; PCI = percutaneous coronary intervention; TIA = transient ischemic attack.

Patients with procedural success and those without did not differ significantly in their baseline characteristics. The baseline echocardiography of patients with procedural success showed increased ventricular size, a lower EF, and poorer right heart function. Most patients in the procedural success group had a reduced EF of less than 50% (69% of patients), whereas most patients in the unsuccessful repair group demonstrated a preserved EF > 50% (88% of patients). (Table 2)

**Table 2:** Baseline echocardiographic characteristics.

Variable (n)	Overall population (n = 40)	No procedural success (n = 8)	Procedural success (n = 32)	P-value
LVEDD – mm (37)	53 ± 9 (37)	49 ± 9 (8)	54 ± 9 (29)	0.20
LVESD – mm (35)	40 ± 12 (35)	33 ± 10 (8)	42 ± 12 (25)	0.06
LVEDV – ml (38)	133 [90–188] (38)	92 [77–156] (8)	141 [90–195] (30)	0.09
LVESV – ml (38)	63 [41–63] (38)	30.5 [20–75] (8)	67.5 [48–127] (30)	0.01
LVEF - % (40)	45 ± 16 (40)	60 ± 13 (8)	41.5 ± 15 (32)	0.03
LVEF ≤ 40%	15 (37.5)	1 (12.5)	14 (44)	
LVEF 41–49%	8 (20)	0	8 (25)	
LVEF > 50%	17 (42.5)	7 (87.5)	10 (31)	
LAVI – ml/m^2^ (37)	67 [53–91] (37)	66.5 [53.25–109] (8)	67 [51–91] (29)	0.87
RVD 1 – mm (37)	50 ± 8 (37)	48 ± 7 (8)	50 ± 8 (29)	0.42
RVD 2 – mm (37)	39 ± 8 (37)	37 ± 7 (8)	39 ± 9 (29)	0.47
TAPSE – mm (33)	16 ± 5 (33)	14 ± 7 (6)	17 ± 4 (27)	0.24
RV FAC - % (35)	34 ± 11 (35)	42 ± 11 (28)	32 ± 10 (7)	0.04
RAA - cm^2^ (37)	36 [30–45]	34 [29–42] (7)	35.5 [30–47] (30)	0.66
TR Vmax – m/s (37)	3.1 ± 0.7 (37)	2.9 ± 0.7 (8)	3.1 ± 0.6 (29)	0.45
Secondary MR – n (%) (40)	34 (85%)	7 (87.5%)	29 (91%)	
MR PISA Radius – mm (19)	9 [6–12] (19)	9 [5.5–13.5] (5)	9 [6–10.5] (14)	1.0
MR EROA - mm^2^ (17)	33.8 ± 27.8 (17)	37 ± 40 (5)	32 ± 23 (12)	0.74
MR Vol – ml (17)	57 [29–93] (17)	39 [22.5–105] (5)	57.5 [35–94] (12)	0.57
MR vena contracta – mm (35)	9 [7–11] (35)	12 [6–15] (7)	8 [7–10] (28)	0.11
MR Inflow Pmean – mm (24)	2.5 [2–3] (24)	3 [3 - 4.3] (5)	2 [2–3] (19)	0.04
MR annular diameter ap x ml – mm (31)	40.0 ± 5.3 x 40.2 ± 4.8 (31)	41 ± 4.5 x 38 ± 3.6 (6)	40 ± 3.6 x 41 ± 5.1 (25)	0.74
Secondary TR - % (40)	100	100	100	
TR EROA - mm^2^ (22)	61 [33–86.5] (22)	50 [27.5–111] (5)	62 [36–87] (17)	0.59
TR Vol – ml (21)	63 ± 24 (21)	54 ± 23 (5)	66 ± 24 (16)	0.31
TR PISA Radius – mm (22)	9.9 ± 3.2 (22)	9.8 ± 2.9 (5)	10.0 ± 3.4 (17)	0.83
TR vena contracta – mm (35)	13 [10–18] (35)	13 [9–19] (7)	13 [10–18] (28)	0.70

Continuous variables given as median [25th-75th percentile] or mean ± standard deviation, and counts as absolute frequencies (column%). Procedural success was defined as reduction of MR ≥ 2° and reduction of TR ≥ 1°.

Abbreviations: EROA = effective regurgitation orifice area; LAVI = left atrial volume index; LVEDD = left ventricular end-diastolic diameter; LVEDV = left ventricular end-diastolic volume; LVEF = left ventricular ejection fraction; LVESV = left ventricular end-systolic volume; LVESD = left ventricular end-systolic diameter; MR = mitral regurgitation; PISA = proximal isovelocity surface area; Pmean = mean pressure gradient; RVD = right ventricular diameter; RAA = right atrial area; RV FAC = right ventricular fractional area change; TAPSE = tricuspid annular plane systolic excursion; TR = tricuspid regurgitation; Vmax = peak velocity

### 3.2. Procedural details

**Fig 1:**
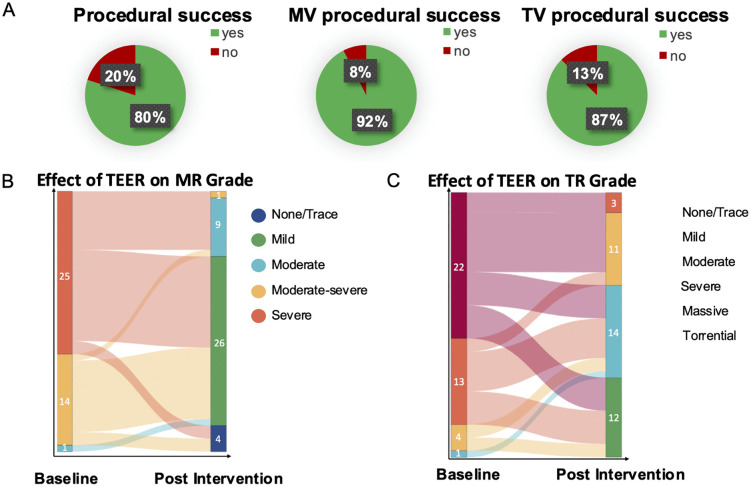
Efficacy of concomitant TEER. **(A)** Pie charts displaying procedural success defined as reduction of MR ≥ 2° and reduction of TR ≥ 1° was achieved in 80% of the treated cases. Procedural success at mitral valve defined as reduction of MR ≥ 2° was achieved in 92% of the cases. Procedural success at tricuspid valve defined as reduction of TR ≥ 1° was achieved in 87% of the cases. (B + **C)** Sankey plot showing the periprocedural transition of MR and TR grade during combined MR/TR TEER. Abbreviations: MR = mitral regurgitation; MV = mitral valve; TEER = transcatheter edge-to-edge repair; TR = tricuspid regurgitation; TV = tricuspid valve.

Procedural success at both valves was reached in 80% of the cases, success at the mitral valve in 92% of all cases, and at the tricuspid valve in 87%. MR was reduced to ≤2° in 98% of the cases and ≤1° in 75%. TR could be decreased to ≤2° in 65% of the cases. The pre- to postinterventional transition of MR and TR is shown in Fig 1. The median procedural time was 137.5 minutes (IQR ± 81). The median number of used devices was significantly higher at the tricuspid vs. the mitral valve (p = 0.048) (Table 3). Most devices at the tricuspid valve were placed between the septal and anterior leaflet (74%), followed by posterior-septal (20%), and anterior-posterior (6%) implantation. Transmitral and transtricuspid gradients were slightly higher after TEER without a clinically meaningful rise. No intra-procedural death, myocardial infarction (MI), stroke or transient ischemic attack (TIA), or severe bleeding occurred, but two patients died during the index hospital stay (in-hospital mortality 5%). One patient died due to a complicated pleural empyema where the valve treatment was done to facilitate a surgical treatment option for the empyema. The other patient died due to severe gastrointestinal bleeding after the procedure and subsequent weaning failure due to nosocomial pneumonia. There was one case of device embolization in which the device was successfully retrieved during the intervention. No case needed conversion to a surgical approach or received surgical treatment within the following year.

**Table 3:** Procedural details.

Variable (n)	Overall population (n = 40)	No procedural success (n = 8)	Procedural success (n = 32)	P-value
Procedural duration – min (40)	137,5 [111–192]	154 [122–248]	133 [107–186.5]	0.31
Length of stay – (40)	9,5 [7–13]	12 [8.0–28]	9 [7–11]	0.10
Length of stay TEER to discharge – days (40)	6,0 [4–7]	6 [5–13]	6 [4–7]	0.22
MV Pmean pre TEER - mmHg (23)	2.6 ± 1 (23)	3 ± 0.8 (4)	2.5 ± 1.1 (19)	0.39
MV Pmean post TEER - mmHg (31)	3 [3–4] (31)	4 [3–4] (7)	3 [3 - 3.75] (24)	0.70
Number of devices Mitral – n (40)	1 (1–2)	1 (1–1.75)	1.5 (1–2)	0.16
Number of devices Tricuspid – n (40)	2 (1–2.75)	1.5 (0–2)	2 (1 –3)	0.13
**Tricuspid devices Position – %**				
anterior-septal	74%	90%	70%	
posterior-septal	20%	10%	25%	
anterior-posterior	6%	0%	5%	
**Intraprocedural Complications**				
Death – n	0	0	0	
MI – n	0	0	0	
Stroke/TIA – n	0	0	0	
Severe bleeding – n	0	0	0	
Device Embolization – n	1	1	0	0.04
Single leaflet attachment – n	0	0	0	
**In Hospital Complications**				
Death – n	2	2	0	0.04
Resuscitation – n	1	1	0	0.04
MI – n	0	0	0	
Stroke/TIA – n	0	0	0	
Severe bleeding – n	2	2	0	0.04
Device Embolization – n	1	1	0	0.04
Single leaflet attachment – n	0	0	0	

Continuous variables given as median [25th-75th percentile] or mean ± standard deviation, and counts as absolute frequencies (column%). Procedural success was defined as reduction of MR ≥ 2° and reduction of TR ≥ 1°.

Abbreviations: MI = myocardial infarction; MV = mitral valve; Pmean = mean pressure gradient; TEER = transcatheter edge-to-edge repair; TIA = transient ischemic attack.

### 3.3. Clinical outcomes

**Fig 2:**
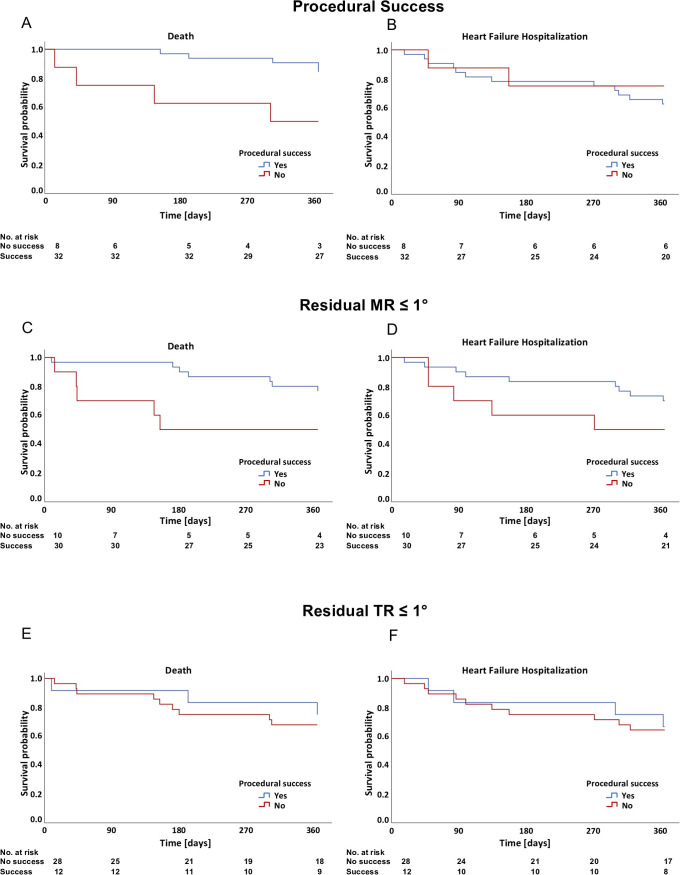
Key outcomes after one year according to procedural success, residual MR and residual TR. Kaplan-Meier analysis of all-cause mortality and heart failure hospitalizations at one year according to (A + B) procedural success (defined as reduction of MR ≥ 2° and reduction of TR ≥ 1, (C + D) according to residual MR and (E + F) according to residual TR. Abbreviations: MR = mitral regurgitation; TR = tricuspid regurgitation.

One year after the intervention, 12 of 40 patients died (30%), and 14 experienced an HFH (35%). In patients with successful repair, mortality rates at one year tended to be lower, with 25% in patients with procedural success vs. 50% in patients without procedural success (Hazard ratio [HR] 0.32 Confidence interval [CI] 0.10, 1.08, Table 4). In patients with residual MR ≤ 1° compared to residual MR ≥ 2°, both mortality rates (23.3% vs. 50%, HR 0.33, CI 0.10, 1.05) and HFH (30% vs. 50%, HR 0.35, CI 0.12, 1.05) tended to be reduced. For residual TR ≤ 1° compared to residual TR ≥ 2°, the differences were less pronounced for 1-year mortality (25% vs. 32.1%, HR 0.70, CI 0.19, 2.57) and HFH (33.3% vs. 35.7% HR 0.83, CI 0.26, 2.65). After adjusting for several markers of adverse outcome, HRs for the different outcomes were consistently reduced as in the unadjusted model (Table 4). Kaplan-Meier curves for the various outcomes are shown in Fig 2. The presence of intracardiac leads did not modify any of the associations between procedural success metrics and clinical outcomes (all p for interaction > 0.36).

**Table 4:** Key outcomes according to procedural success, residual MR ≤ 1°, and residual TR ≤ 1°.

	Outcome	Events in patients without achieved result	Events in patients with achieved result	HR (95% CI), P-value	Adjusted HR (95% CI), P-value
**Procedural success**	death in first year	4 (50.0%)	8 (25.0%)	0.32 95% CI (0.10, 1.08) p = 0.066	0.38 95% CI (0.08, 1.74) p = 0.211
	heart failure hospitalization in first year	2 (25.0%)	12 (37.5%)	1.07 95% CI (0.24, 4.80) p = 0.926	1.06 95% CI (0.17, 6.71) p = 0.952
**Residual MR ≤ 1**	death in first year	5 (50.0%)	7 (23.3%)	0.33 95% CI (0.10, 1.05) p = 0.060	0.41 95% CI (0.11, 1.54) p = 0.185
	heart failure hospitalization in first year	5 (50.0%)	9 (30.0%)	0.35 95% CI (0.12, 1.05) p = 0.061	0.18 95% CI (0.05, 0.62) p = 0.007
**Residual TR ≤ 1**	death in first year	9 (32.1%)	3 (25.0%)	0.70 95% CI (0.19, 2.57) p = 0.587	0.77 95% CI (0.18, 3.25) p = 0.726
	heart failure hospitalization in first year	10 (35.7%)	4 (33.3%)	0.83 95% CI (0.26, 2.65) p = 0.754	0.82 95% CI (0.24, 2.84) p = 0.760

Procedural success was defined as reduction of MR ≥ 2° and reduction of TR ≥ 1°.

Abbreviations: CI = confidence interval; HR = hazard ratio; MR = mitral regurgitation; TR = tricuspid regurgitation.

In patients with available NYHA status at follow-up (15 patients, i.e., 53% of patients alive at one year), NYHA status was improved compared to baseline with 13% NYHA I, 67% NYHA II, and 20% NYHA III compared with 13% NYHA II, 67% NYHA III, and 20% NYHA IV at baseline (p = 0.001, central illustration).

## 4. Discussion

In this retrospective, hypothesis-generating study, we performed a cross-sectional analysis of 40 patients treated with concurrent MR/TR TEER for severe MR and TR. We demonstrated that a simultaneous treatment approach was safe in our cohort and effectively reduced MR and TR in most treated patients. Patients receiving successful repair of both valves showed a consistent trend towards reduced one-year mortality and HFH compared to those without successful repair. This association remained after adjusting for several parameters of adverse outcomes.

The number of TEER procedures has dramatically increased in the past years, partly driven by the positive results of the COAPT trial. In this trial, treatment with TEER for severe secondary MR in patients with heart failure on guideline-directed medical therapy (GDMT) showed a marked reduction in HFH and mortality rates [3]. In contrast, in the Mitra.FR trial, patients with severe secondary MR and HF did not benefit from an M-TEER procedure [11]. A concept trying to explain the different results is the concept of proportionate and disproportionate MR. Briefly, the idea is that patients with MR that is more significant than one would predict by the degree of left ventricular dilatation would benefit most from a treatment of the MR [12]. Data from the EuroSMR registry retrospectively assigning more than 1600 patients to a COAPT or MITRA.FR eligible versus non-eligible cases confirmed the mortality reduction observed in the COAPT study for COAPT eligible but not MITRA-FR eligible cases while showing symptomatic benefit for all groups [13]. Most recently, the RESHAPE-2 trial investigating M-TEER by MitraClip implantation in a collective of HF patients with moderate to high-grade MR met its primary endpoint consisting of HFH and CV death. It recruited HF patients with left ventricular diameters comparable to the COAPT collective but considerably less MR than in both COAPT and even MITRA.FR (EROA 0.23 cm^2^ vs. 0.40 cm^2^ vs. 0.31 cm^2^). A striking reduction in HFHs mainly drove the primary results [14]. These data suggest that it is the degree of left heart disease that may decide whether MR intervention is merely symptomatic or an outcome-relevant procedure. Among those with relatively preserved left ventricular geometry and function, the degree of MR may determine whether HFH or mortality is reduced within a given time frame [15]. While most data was generated employing the MitraClip device, the recent CLASP IID trial suggested comparable safety and efficacy for the Pascal device for the treatment of MR [16].

For T-TEER data from the randomized controlled TRILUMINATE trial, assigning patients with symptomatic severe TR to T-TEER on top of GDMT vs. GDMT alone are now available [4]. The trial met its primary endpoint, a hierarchical composite that included death from any cause, HFH, and quality of life improvement measured by the Kansas City Cardiomyopathy Questionnaire (KCCQ). Improvement in quality of life, as indicated by the KCCQ, was the driving factor behind the positive results, although the effects on cardiovascular outcomes and the distance in the 6-minute walking test (6MWT) were neutral. The trial also strengthened the evidence of the safety of T-TEER, reporting a 30-day cardiovascular mortality rate of 1.7% in the interventional group, with no myocardial infarction (MI), no stroke, a new-onset renal failure rate of 1.2%, major bleeding at 4.7%, and single leaflet device attachment at 7%. These results have been recently corroborated in a very similar cohort within the TRI.Fr trial, an investigator-initiated, industry-independent trial [17].

In our study, intrahospital rates of major adverse events related to the procedure scored 5% for intrahospital death, 0% for MI or stroke, and 5% for bleeding, so there is no sign of excess complications when combining the procedures. These data align with Mehr and colleagues’ publication, including patients (n = 122) with combined TEER for MR and TR from the TRAMI and TriValve registries [18]. Here, complication rates with concomitant TEER were 4.1% for intrahospital death, 1.6% for stroke, and no cases of MI. Interestingly, complication rates were comparable to those of 106 patients who received M-TEER alone. Therefore, combination therapy might be an option to increase patient comfort and safety without needing another hospitalization and procedure. Compared to sequential TEER procedures, avoiding a second vascular access and general anaesthesia/sedation may translate into a reduced risk of nosocomial infection or delirium. These complications were shown to be as high as 7.2% for infection and 2.6% for delirium in patients treated with M-TEER and admitted to the intensive care unit, which can lead to higher morbidity and mortality and thus should be prevented as best as possible [19]. Recent analyses report a 5–6 times higher mortality rate of patients acquiring nosocomial infections of absolute values up to 30% [20]. It should be noted that the feasibility of combined mitral and tricuspid TEER may also be influenced by non-clinical factors such as reimbursement regulations, which in some healthcare systems do not support concomitant transcatheter valve interventions despite potential clinical benefit. However, if randomized controlled trials were to demonstrate a clear clinical advantage of a combined approach, this could help inform future reimbursement policies and facilitate broader adoption.

The prognostic benefit of TEER for MR or TR in different patient populations remains uncertain. As stated before, a beneficial effect on mortality or HFH has only been shown in selected patients with moderate-to-severe or severe secondary MR and HF in the RESHAPE2 and COAPT trial. The five-year follow-up of COAPT recently showed a persistent reduction of all-cause mortality for patients treated with TEER (for mortality 57.3% in TEER + GDMT vs. 67.2% in GDMT alone) [21]. However, in the Mitra.FR trial, patients with severe MR and progressed HF did not benefit either in mortality or HFH after 12 months (for mortality 24.3% in TEER + GDMT vs. 22.4% in GDMT alone) [11]. Between 12 and 24 months of follow up the frequency of HFH was halved in the treatment versus GDMT group but did not reach statistical significance due to low numbers [22]. Also, for T-TEER, in the TRILUMINATE trial, no difference in one-year mortality or HFH was observed [23]. Those data indicate that distinct patient populations respond differently to TEER treatment and deserve dedicated consideration in clinical trials. Compared to the established trials on isolated M-TEER and T-TEER, our findings suggest a potential mortality benefit of combined TEER in a selected high-risk cohort. While COAPT demonstrated reductions in both mortality and HF hospitalizations, MITRA-FR did not show benefit, and RESHAPE-HF2 was primarily driven by hospitalization reduction [3, 4, 11]. TRILUMINATE showed quality-of-life improvement without an effect on hard clinical endpoints [23]. In contrast, our data indicate that addressing both MR and TR in a single session may provide additive benefit. In a previous study by Besler and colleagues, sixty-one patients with significant MR and TR were treated either with M-TEER (n = 34) or combined M- and T-TEER (n = 27). In their study, the combined treatment led to superior improvement in functional parameters (NYHA, 6MWT, NT-proBNP levels) and HFH, while rates of death were comparable [7]. However, in the analysis from the TRAMI and TriValve registries by Mehr and colleagues, combined treatment of MR and TR associated with a lower rate of 1-year mortality compared to patients treated only for MR in case of coexisting significant TR (16.4% 1-year mortality in combined treatment vs. 34% in M-TEER treated), which is in line with the findings of our study [18].

In patients with severe TR and MR, the chance of spontaneous recovery after M-TEER is low. In the largest multicentre study evaluating the evolution of TR after M-TEER for functional MR, a reduction of TR ≥ 1° was reported in 35% of the cases [6]. However, at baseline, 27% of all patients had severe TR (grade 3 or 4), and at short-term follow-up, 26% still had severe TR. These data demonstrate that a significant TR reduction in those patients cannot be expected. In addition, in patients with a baseline TR graded severe or worse, a decrease of one or two grades would still leave them with a moderate TR or worse. Therefore, a simultaneous treatment of TR seems not only feasible but reasonable. Another point to consider is that different studies have shown that advanced right heart disease (right heart failure, more severe TR) affords worse outcomes after TEER [24–26]. This has led to the concept that earlier intervention might offer better outcomes in affected patients. Suppose a patient with MR and TR is treated only for MR. In that case, he/she might already feel a decent amount of symptomatic benefit and therefore be reluctant to undergo another intervention, thus likely does not present again unless right heart disease has progressed to a stage where intervention might be less favourable.

Importantly, the decision toward a combined M- and T-TEER treatment has to be based on pulmonary pressure, pulmonary vascular resistance, right heart function and classification of tricuspid regurgitation. Patient with high pulmonary pressure and decreased right ventricular function, which is referred as right ventricular uncoupling, are at high risk for worse outcome and might benefit from a staged approach. Sisinni and colleagues defined three options for patients undergoing M-TEER and have a concomitant tricuspid regurgitation based on the clinical scenario. Patients with a fixed primary pulmonary hypertension, might better not undergo T-TEER, patients with postcapillary pulmonary hypertension and a reduced right ventricular function, may benefit from a staged approach, and patients without those features might directly undergo combined procedures [27].

Furthermore, current expert statements regarding patients with pacemaker-lead associated tricuspid regurgitation should be considered in the decision-making process, as those patients are not likely to improve with M-TEER alone [28].

## 5. Limitations

Limitations of our study include potential bias due to its retrospective nature. Due to its cross-sectional design, associations cannot be directly attributed to causality. Also, the single-centre design precludes the generalizability of results. Given the limited sample size, the study was underpowered for clinical endpoints such as mortality. Multiple subgroup analyses were performed without formal multiplicity adjustment; therefore, all inferential results should be considered exploratory. The COVID-19 pandemic affected follow-up times, so not all patients were comprehensively evaluated one year after intervention. However, data regarding mortality are very robust since they were acquired via Statistic Austria, a national database tracking all Austrian deaths, and diligence was exercised in blinding all data before analysis and interpretation.

## 6. Conclusion

Our findings provide additional evidence corroborating the notion that combined TEER of severe MR and TR is not only feasible, significantly reducing MR and TR, but may also be associated with better outcomes. These data warrant an RCT to provide pertinent answers to this question.

## 7. Impact on daily practice

Simultaneous TEER of mitral and tricuspid regurgitation appears to be a feasible and safe treatment option for selected patients with advanced heart failure and dual-valve disease. In our cohort, this approach was associated with favourable procedural outcomes and signals toward improved clinical status. These findings may support considering combined TEER in carefully selected cases, pending confirmation in larger prospective studies.

## Supporting information

S1 Table Baseline characteristics split by residual MR. Continuous variables given as median [25th-75th percentile] or mean ± standard deviation, and counts as absolute frequencies (column%).
(PDF)

S2 Table Baseline characteristics split by residual TR. Continuous variables given as median [25th-75th percentile] or mean ± standard deviation, and counts as absolute frequencies (column%).
(PDF)

S3 Table Baseline echocardiographic characteristics split by residual MR. Continuous variables given as median [25th-75th percentile] or mean ± standard deviation, and counts as absolute frequencies (column%).
(PDF)

S4 Table Baseline echocardiographic characteristics split by residual TR. Continuous variables given as median [25th-75th percentile] or mean ± standard deviation, and counts as absolute frequencies (column%).
(PDF)

S1 Fig Kaplan-Meier curves displaying survival probability for different procedural success definitions. (a) procedural success defined as reduction of MR & TR ≥ 2°, (b) procedural success defined as residual MR ≤ 1° & TR ≤ 2°.
(PDF)

S5 Table Key outcomes according to reduction of MR & TR ≥ 2°. (PDF)

S6 Table Key outcomes according to residual MR ≤ 1° & TR ≤ 2°. (PDF)

S1 Minimal dataset Anonymized raw data underlying the results of the study, including baseline characteristics, procedural variables, echocardiographic parameters, and follow-up outcomes used for statistical analyses and figure generation. (XLSX)
