# Supplementary material for: Simultaneous transcatheter edge-to-edge repair (TEER) for severe mitral and tricuspid regurgitation is feasible, safe, and associated with good clinical outcome
Source: PLoS One. 2026 Feb 10;21(2):e0339837. doi: 10.1371/journal.pone.0339837 (PMC12890156; doi:10.1371/journal.pone.0339837)
Supplement: S1 Table — Continuous variables given as median [25th-75th percentile] or mean ± standard deviation, and counts as absolute frequencies (column%). (PDF) [file pone.0339837.s001.pdf]

Supplementary table 1: Baseline characteristics split by residual MR

Variable	Overall population (n=40)	Residual MR ≤ 1° (n=30)	Residual MR ≥ 2° (n=10)	P-value
Age at procedure - years	78 (73 - 83)	78 (73 – 82)	78.5 (74 – 86)	0.51
Female sex - n (%)	23 (57.5)	18 (60)	5 (50)	0.58
Height - cm	169 ± 8	169 ± 8	168 ± 5	0.58
Weight - kg	70 ± 13	69 ± 13	72 ± 14	0.61
BMI - kg/m^2	25 ± 4	24 ± 4	26 ± 4	0.34
NYHA - n (%)				0.95
II	8 (20)	6 (20)	2 (20)	0.58
III	29 (72.5)	21 (70)	8 (80)	
IV	3 (7.5)	3 (10)	0	
Comorbidities				
Ischemic Heart Disease - n (%)	16 (40)	12 (40)	4 (30)	0.92
Dilated Cardiomyopathy – n (%)	13 (32.5)	10 (33)	3 (30)	0.97
Diabetes mellitus – n (%)	8 (20)	5 (17)	3 (30)	0.36
Arterial Hypertension – n (%)	33 (82.5)	25 (83)	8 (80)	0.81
Hypercholesterinemia – n (%)	15 (37.5)	11 (37)	4 (40)	0.85
COPD – n (%)	8 (20)	8 (12.5)	0	0.07
CKD (≥ IIIa) – n (%)	33 (82.5)	23 (74)	10 (100)	0.36
Atrial fibrillation/flutter – n (%)	37 (92.5)	28 (93)	9 (90)	0.72
Pulmonary Hypertension – n (%)	15 (37.5)	10 (33)	5 (50)	0.34
PAD – n (%)	6 (15)	4 (13)	2 (20)	0.61
Cerebrovascular artery disease – n (%)	1 (2.5)	0	1 (10)	0.08
Previous myocardial infarction – n (%)	5 (12.5)	5 (17)	0	0.17
Previous PCI – n (%)	12 (30)	10 (33)	2 (20)	0.42
Previous CABG – n (%)	4 (10)	3 (10)	1 (10)	1.0
Previous valve surgery – n (%)	4 (10)	3 (10)	1 (10)	0.73
Implanted intracardiac Device - n (%)	12 (30)	9 (30)	3 (43)	0.55
Pacemaker - n (%)	7 (17.5)	6 (12.5)	1 (10)	N/A
ICD - n (%)	4 (10)	2 (6.7)	2 (20)	N/A
CRT - n (%)	1 (2.5)	1 (3)	0	N/A
Previous Stroke/TIA - n (%)	3 (7.5)	3 (10)	0	0.30
Baseline Laboratory values				

Creatinine - mg/dl	1.35 (1.09 – 1.73)	1.24 (1.04 – 2.72)	1.44 (1.29 – 1.85)	0.30
Hemoglobin - g/dl	12.1 ± 1.7	12.3 ± 1.5	11.5 ± 2.9	0.23
NT-proBNP - pg/ml	3891 (2356 – 9442)	6308 (2492– 13817)	3168 (1206 – 5305)	0.10
hsTroponinT - pg/ml	37 (19.75 – 67.75)	37 (18.5 – 70)	42 (22 – 63.26)	0.58

Continuous variables given as median [25th-75th percentile] or mean ± standard deviation, and counts as absolute frequencies (column%).

Abbreviations: BMI=body mass index; CABG=coronary artery bypass graft; CKD=chronic kideney disease; cm=centimetre; COPD=chronic obstructive pulmonary disease; CRT=cardiac resynchronization therapy; ICD=implantable cardioverter defibrillator; kg=kilogram; NT-proBNP=N-terminal pro-B-type natriuretic peptide; NYHA=New York Heart Association; PAD=peripheral arterial disease; PCI=percutaneous coronary intervention; TIA=transient ischemic attack.
