# Supplementary material for: Simultaneous transcatheter edge-to-edge repair (TEER) for severe mitral and tricuspid regurgitation is feasible, safe, and associated with good clinical outcome
Source: PLoS One. 2026 Feb 10;21(2):e0339837. doi: 10.1371/journal.pone.0339837 (PMC12890156; doi:10.1371/journal.pone.0339837)
Supplement: S2 Table — Continuous variables given as median [25th-75th percentile] or mean ± standard deviation, and counts as absolute frequencies (column%). (PDF) [file pone.0339837.s002.pdf]

Supplementary table 2: Baseline characteristics split by residual TR

Variable	Overall population (n=40)	Residual TR° ≤ 1 (n=12)	Residual TR° ≥ 2 (n=28)	P-value
Age at procedure - years	78 (73 - 83)	73 (70 – 80)	79.5 (76 – 83)	0.07
Female sex – n (%)	23 (57.5 %)	7 (58 %)	16 (57 %)	0.58
Height [cm ± SD]	169 ± 8	168 ± 6.5	168 ± 6.5	0.85
Weight [kg ± SD]	70 ± 13	74 ± 13	74 ± 13	0.24
BMI [kg/m² ± SD]	25 ± 4	26 ± 3.7	26 ± 3.7	0.15
NYHA – n (%)				0.27
II	8 (20)	3 (25)	5 (18)	0.58
III	29 (72.5)	7 (58)	22 (79)	
IV	3 (7.5)	2 (17)	1 (4)	
Comorbidities				
Ischemic Heart Disease – n (%)	16 (40)	8 (67)	8 (29)	0.08
Dilated Cardiomyopathy – n (%)	13 (32.5)	2 (17)	11 (39)	0.24
Diabetes mellitus – n (%)	8 (20)	3 (25)	5 (18)	0.61
Arterial Hypertension – n (%)	33 (82.5)	10 (83)	23 (82)	0.93
Hypercholesterinemia – n (%)	15 (37.5)	6 (50)	9 (32)	0.29
COPD – n (%)	8 (20)	2 (17)	6 (21)	0.73
CKD (≥ IIIa) – n (%)	33 (82.5)	10 (83)	23 (82)	0.86
Atrial fibrillation/flutter – n (%)	37 (92.5)	11 (92)	26 (93)	0.90
Pulmonary Hypertension – n (%)	15 (37.5)	6 (50)	9 (32)	0.29
PAD – n (%)	6 (15)	4 (33)	2 (7)	0.03
Cerebrovascular artery disease – n (%)	1 (2.5)	1 (8)	0	0.12
Previous myocardial infarction – n (%)	5 (12.5)	2 (17)	3 (11)	0.60
Previous PCI – n (%)	12 (30)	6 (50)	6 (21)	0.07
Previous CABG – n (%)	4 (10)	4 (33)	0	0.001
Previous valve surgery – n (%)	4 (10)	3 (25)	1 (4)	N/A
Implanted intracardiac Device – n (%)	12 (30)	5 (42)	7 (25)	0.20
Pacemaker – n (%)	7 (17.5)	2 (17)	5 (18)	N/A
ICD – n (%)	4 (10)	3 (25)	1 (4)	N/A
CRT – n (%)	1 (2.5)	0	1 (4)	N/A
Previous Stroke/TIA – n (%)	3 (7.5)	1 (8)	2 (7)	0.90
Baseline Laboratory values				

Creatinine - mg/dl	1.35 (1.09 – 1.73)	1.39 (1.18 – 1.76)	1.35(1.04 – 1.65)	0.41
Hemoglobin - g/dl	12.1 ± 1.7	12.3 ± 2.3	12.3 ± 2.3	0.66
NT-proBNP - pg/ml	3891 (2356 – 9442)	3397 (1411– 8922)	4281 (2471 – 12538)	0.44
hsTroponinT - pg/ml	37 (19.75 – 67.75)	36 (18.3 – 67.8)	37 (20 – 73)	0.69

Continuous variables given as median [25th-75th percentile] or mean ± standard deviation, and counts as absolute frequencies (column%).

Abbreviations: BMI=body mass index; CABG=coronary artery bypass graft; CKD=chronic kideney disease; cm=centimetre; COPD=chronic obstructive pulmonary disease; CRT=cardiac resynchronization therapy; ICD=implantable cardioverter defibrillator; kg=kilogram; NT-proBNP=N-terminal pro-B-type natriuretic peptide; NYHA=New York Heart Association; PAD=peripheral arterial disease; PCI=percutaneous coronary intervention; TIA=transient ischemic attack.
