# Supplementary material for: Simultaneous transcatheter edge-to-edge repair (TEER) for severe mitral and tricuspid regurgitation is feasible, safe, and associated with good clinical outcome
Source: PLoS One. 2026 Feb 10;21(2):e0339837. doi: 10.1371/journal.pone.0339837 (PMC12890156; doi:10.1371/journal.pone.0339837)
Supplement: S3 Table — Continuous variables given as median [25th-75th percentile] or mean ± standard deviation, and counts as absolute frequencies (column%). (PDF) [file pone.0339837.s003.pdf]

Supplementary table 3: Baseline echocardiographic characteristics split by residual MR

Variable (n)	Overall population (n=40)	Residual MR ≤ 1° (n=30)	Residual MR ≥ 2° (n=10)	P-value
LVEDD - mm(37)	53 ± 9	53 ± 9	52 ± 8	0.84
LVESD - mm (35)	40 ± 12	41 ± 12	37 ± 11	0.39
LVEDV - ml (38)	133 (90 - 188)	144 (89 – 189)	100 (88 - 154)	0.39
LVESD -ml (38)	63 (41 - 63)	73 (43 – 135)	50 (31 - 70)	0.11
LVEF - % (40)	45 ± 16	42 ± 16	53 ± 14	0.06
LVEF ≤ 40%	15 (37.5)	13 (43)	2 (20)	
LVEF 41-49%	8 (20)	5 (17)	3 (30)	
LVEF > 50%	17 (42.5)	12 (40)	5 (50)	
LAVI - ml/m^2 (37)	67 (53 - 91)	68 (53 - 88)	60 (47 - 144)	0.93
RVD 1 - mm (37)	50 ± 8	51 ± 8	47 ± 6	0.14
RVD 2 - mm (37)	39 ± 8	41 ± 9	34 ± 3	0.02
TAPSE - mm (33)	16 ± 5	16 ± 5	16 ± 4	0.94
RV FAC - % (35)	34 ± 11	33 ± 10	40 ± 11	0.09
RAA - cm^2 (37)	36 (30 - 45)	36 (32 – 49)	32 (26 - 39)	0.09
TR Vmax - m/s (37)	3.1 ± 0.7	3.0 ± 0.7	3.2 ± 0.7	0.42
Secondary MR - n (%) (40)	34 (85)	27 (90)	7 (70)	
MR PISA Radius - mm (19)	9 (6 – 12)	8.5 (6 – 10.5)	9 (7 – 16)	0.50
MR EROA - mm^2 (17)	33.8 ± 27.8	33 ± 24	34 ± 39	0.97
MR Vol - ml (17)	57 (29 – 93)	55 (28 - 86)	57 (29 - 152)	0.72
MR vena contracta - mm (35)	9 (7 – 11)	8 (7 - 10)	10 (8 - 14)	0.14
MR Inflow Pmean - mm (24)	2.5 (2.0 – 3)	2 (2 – 3.3)	3 (2 – 3)	0.49
MR annular diameter ap x ml - mm (31)	40.0 ± 5.3 x 40.2 ± 4.8	40 ± 5.3 x 41 ± 5.1	41 ± 5.5 x 39 ± 4	0.59
Secondary TR - %	100	100	100	
TR EROA - mm^2 (22)	61 (33 – 86.5)	63 (35 – 84)	57 (29 - 139)	0.97
TR Vol - ml (21)	63 ± 24	63 ± 21	64 ± 33	0.99
TR PISA Radius - mm (22)	9.9 ± 3.2	9.5 ± 3.1	11 ± 3.5	0.32
TR vena contracta - mm (35)	13 (10 – 18)	13 (10 - 18)	13 (8 - 19)	0.64

Continuous variables given as median [25th-75th percentile] or mean ± standard deviation, and counts as absolute frequencies (column%).

Abbreviations: EROA=effective regurgitation orifice are; LAVI=left atrial volume index; LVEDD=left ventricular enddiastolic diameter; LVEDV=left ventricular enddiasstolic volume;

LVEF=left ventricular ejection fraction; LVESV=left ventricular endsystolic volume; LVESD=left ventricular endsystolic diameter; MR=mitral regurgitation; PISA=proximal isovelocity surface area; Pmean=mean pressure gradient; RVD=right ventricular diameter; RAA=right atrial area; RV FAC=right ventricular fractional area change; TAPSE=tricuspid annular plane systolic excursion; TR=tricuspid regurgitation; Vmax=peak velocity.
