# Supplementary material for: Simultaneous transcatheter edge-to-edge repair (TEER) for severe mitral and tricuspid regurgitation is feasible, safe, and associated with good clinical outcome
Source: PLoS One. 2026 Feb 10;21(2):e0339837. doi: 10.1371/journal.pone.0339837 (PMC12890156; doi:10.1371/journal.pone.0339837)
Supplement: S4 Table — Continuous variables given as median [25th-75th percentile] or mean ± standard deviation, and counts as absolute frequencies (column%). (PDF) [file pone.0339837.s004.pdf]

Supplementary table 4: Baseline echocardiographic characteristics split by residual TR.

Variable (n)	Overall population (n=40)	Residual TR ≤ 1° (n=12)	Residual TR ≥ 2° (n=28)	P-value
LVEDD - mm (37)	53 ± 9	52 ± 10	53 ± 8	0.75
LVESD - mm (35)	40 ± 12	39 ± 15	40 ± 10	0.87
LVEDV - ml (38)	133 (90 - 188)	134 (90 - 229)	132 (87 - 176)	0.70
LVESD - ml (38)	63 (41 - 63)	60 (43 - 129)	63 (34 - 113)	0.74
LVEF - % (40)	45 ± 16	43 ± 14	46 ± 17	0.57
LVEF ≤ 40%	15 (37.5)	6 (59)	9 (32)	
LVEF 41-49%	8 (20)	2 (17)	6 (21)	
LVEF ≥ 50%	17 (42.5)	4 (33)	13 (46)	
LAVI - ml/m^2 (37)	67 (53 - 91)	63 (48 - 80)	68 (53 - 102)	0.45
RVD 1 - mm (37)	50 ± 8	48 ± 10	51 ± 7	0.42
RVD 2 - mm (37)	39 ± 8	35 ± 7	41 ± 8	0.04
TAPSE – mm (33)	16 ± 5	16 ± 4	16 ± 5	0.68
RV FAC - % (35)	34 ± 11	34 ± 13	34 ± 10	0.92
RAA - cm^2 (37)	36 (30 - 45)	32 (29 - 36)	37 (31 - 49)	0.04
TR Vmax - m/s (37)	3.1 ± 0.7	3.4 ± 0.5	2.9 ± 1	0.03
Secondary MR - n (%) (40)	34 (85)	10 (83)	24 (86)	
MR PISA Radius - mm (19)	9 (6 – 12)	8 (6 - 11)	9 (7 - 12)	0.37
MR EROA - mm^2 (17)	33.8 ± 27.8	30 ± 32	35 ± 27	0.73
MR Vol – ml (17)	57 (29 – 93)	42 (23 - 90)	58 (33 - 95)	0.44
MR vena contracta - mm (35)	9 (7 – 11)	8 (7 - 10)	8 (7 - 10)	0.15
MR Inflow Pmean - mm (24)	2.5 (2.0 – 3)	2 (1 - 3)	2 (1 - 3)	0.08
MR annular diameter ap x ml - mm (31)	40.0 ± 5.3 x 40.2 ± 4.8	37 ± 4 x 37 ± 4	42 ± 5 x 41 ± 5	0.02
Secondary TR – % (40)	100	100	100	
TR EROA - mm^2 (22)	61 (33 – 86.5)	46 (33 - 70)	46 (33 - 70)	0.26
TR Vol - ml (21)	63 ± 24	54 ± 18	69 ± 26	0.20
TR PISA Radius - mm (22)	9.9 ± 3.2	10 ± 2	10 ± 4	0.89
TR vena contracta - mm (35)	13 (10 – 18)	11 (8 - 13)	14 (10 - 20)	0.01

Continuous variables given as median [25th-75th percentile] or mean ± standard deviation, and counts as absolute frequencies (column%).

Abbreviations: EROA=effective regurgitation orifice are; LAVI=left atrial volume index; LVEDD=left ventricular enddiastolic diameter; LVEDV=left ventricular enddiasstolic volume; LVEF=left ventricular ejection fraction; LVESV=left ventricular endsystolic volume; LVESD=left ventricular endsystolic diameter; MR=mitral regurgitation; PISA=proximal isovelocity surface area; Pmean=mean pressure gradient; RVD=right ventricular diameter; RAA=right atrial area; RV FAC=right ventricular fractional area change; TAPSE=tricuspid annular plane systolic excursion; TR=tricuspid regurgitation; Vmax=peak velocity.
