# Supplementary figures and images for: Simultaneous transcatheter edge-to-edge repair (TEER) for severe mitral and tricuspid regurgitation is feasible, safe, and associated with good clinical outcome
Source: PLoS One. 2026 Feb 10;21(2):e0339837. doi: 10.1371/journal.pone.0339837 (PMC12890156; doi:10.1371/journal.pone.0339837)

# Death

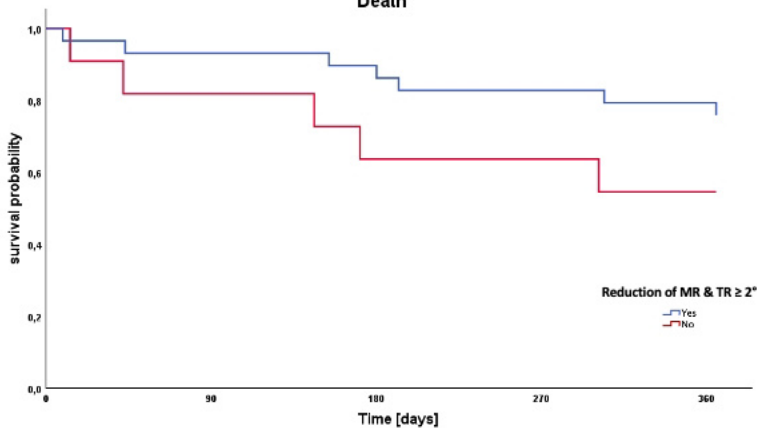

Numbers at risk

No success

Success

11

29

9

27

7

25

6

23

6

22

# Death

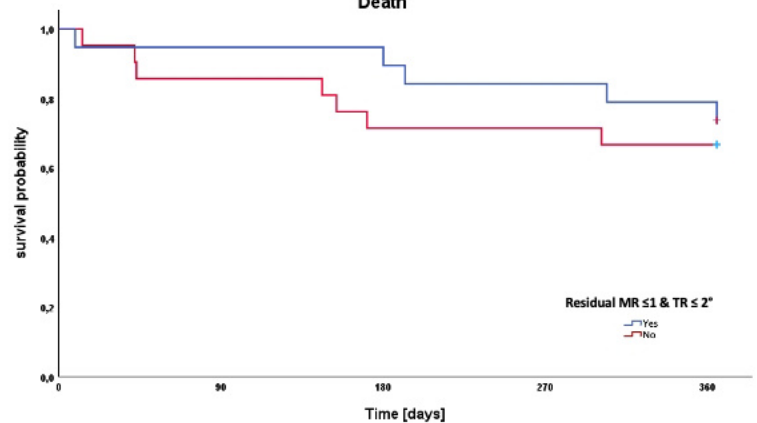

Numbers at risk

No success

Success

11

29

9

27

7

25

6

23

6

22

Supplement: S1 Fig — (a) procedural success defined as reduction of MR & TR ≥ 2°, (b) procedural success defined as residual MR ≤ 1° & TR ≤ 2°. (PDF) [file pone.0339837.s005.pdf]
