# Supplementary material for: Simultaneous transcatheter edge-to-edge repair (TEER) for severe mitral and tricuspid regurgitation is feasible, safe, and associated with good clinical outcome
Source: PLoS One. 2026 Feb 10;21(2):e0339837. doi: 10.1371/journal.pone.0339837 (PMC12890156; doi:10.1371/journal.pone.0339837)
Supplement: S5 Table — (PDF) [file pone.0339837.s006.pdf]

Supplementary table 6: Key outcomes according to reduction of MR & TR grade ≥ 2°.

Outcome	Events in Patients without Reduction of MR & TR grade ≥ 2°	Events in Patients with Reduction of MR & TR grade ≥ 2°	Unadjusted HR (95% CI), P-value	Adjusted HR (95% CI), P-value
death in year 1	5 (45.5%)	7 (24.1%)	0.41 95% CI (0.13 ,1.28) p = 0.124	0.34 95% CI (0.08 ,1.45) p = 0.144
heart failure hospitalization in first year	3 (27.3%)	11 (37.9%)	1.21 95% CI (0.34 ,4.33) p = 0.772	1.02 95% CI (0.20 ,5.10) p = 0.983

Procedural success was defined as reduction of MR ≥ 2° and reduction of TR ≥2°.
Abbreviations: CI=confidence interval; HR=hazard ratio; MR=mitral regurgitation; TR=tricuspid regurgitation
