# Supplementary material for: Simultaneous transcatheter edge-to-edge repair (TEER) for severe mitral and tricuspid regurgitation is feasible, safe, and associated with good clinical outcome
Source: PLoS One. 2026 Feb 10;21(2):e0339837. doi: 10.1371/journal.pone.0339837 (PMC12890156; doi:10.1371/journal.pone.0339837)
Supplement: S6 Table — (PDF) [file pone.0339837.s007.pdf]

Supplementary table 7: Key outcomes according to residual MR ≤ 1° & TR ≤ 2°.

Outcome	Events in Patients without residual MR ≤ 1 & TR ≤ 2°	Events in Patients with residual MR ≤ 1 & TR ≤ 2°	Unadjusted HR (95% CI), P-value	Adjusted HR (95% CI), P-value
death in year 1	7 (33.3%)	5 (26.3%)	0.69 95% CI (0.22 ,2.16) p = 0.519	0.94 95% CI (0.28 ,3.14) p = 0.918
heart failure hospitalization in first year	9 (42.9%)	5 (26.3%)	0.47 95% CI (0.16 ,1.40) p = 0.175	0.31 95% CI (0.09 ,0.99) p = 0.049

Procedural success was defined as residual MR ≤ 1° and residual TR ≤2°.
Abbreviations: CI=confidence interval; HR=hazard ratio; MR=mitral regurgitation; TR=tricuspid regurgitation
